# Cloud-based uniform ChIP-Seq processing tools for modENCODE and ENCODE

**DOI:** 10.1186/1471-2164-14-494

**Published:** 2013-07-22

**Authors:** Quang M Trinh, Fei-Yang Arthur Jen, Ziru Zhou, Kar Ming Chu, Marc D Perry, Ellen T Kephart, Sergio Contrino, Peter Ruzanov, Lincoln D Stein

**Affiliations:** 1 Ontario Institute for Cancer Research, MaRS Centre, South Tower, 101 College Street, Suite 800, Toronto, ON, M5G 0A3, Canada; 2 Department of Genetics, University of Cambridge, Downing Street, Cambridge, CB2 3EH, UK; 3 Department of Molecular Genetics, University of Toronto, 1 Kings College Circle, Toronto, ON, M5S 1A8, Canada

## Abstract

**Background:**

Funded by the National Institutes of Health (NIH), the aim of the **Mod**el Organism **ENC**yclopedia **o**f **D**NA **E**lements (modENCODE) project is to provide the biological research community with a comprehensive encyclopedia of functional genomic elements for both model organisms C. elegans (worm) and D. melanogaster (fly). With a total size of just under 10 terabytes of data collected and released to the public, one of the challenges faced by researchers is to extract biologically meaningful knowledge from this large data set. While the basic quality control, pre-processing, and analysis of the data has already been performed by members of the modENCODE consortium, many researchers will wish to reinterpret the data set using modifications and enhancements of the original protocols, or combine modENCODE data with other data sets. Unfortunately this can be a time consuming and logistically challenging proposition.

**Results:**

In recognition of this challenge, the modENCODE DCC has released uniform computing resources for analyzing modENCODE data on Galaxy (https://github.com/modENCODE-DCC/Galaxy), on the public Amazon Cloud (http://aws.amazon.com), and on the private Bionimbus Cloud for genomic research (http://www.bionimbus.org). In particular, we have released Galaxy workflows for interpreting ChIP-seq data which use the same quality control (QC) and peak calling standards adopted by the modENCODE and ENCODE communities. For convenience of use, we have created Amazon and Bionimbus Cloud machine images containing Galaxy along with all the modENCODE data, software and other dependencies.

**Conclusions:**

Using these resources provides a framework for running consistent and reproducible analyses on modENCODE data, ultimately allowing researchers to use more of their time using modENCODE data, and less time moving it around.

## Background

As an integral part of the modENCODE project (https://www.genome.gov/modENCODE/), the primary role of the DCC has been to collect, validate, and release data from the 11 sub-projects. This is a challenge; not only does the data span a wide range of data types ranging from chromatin profiling to gene model annotation [1,2], but a large variety of methods and platforms were used to collect and analyze the data over the project’s five year life span, including several changes to the reference genome builds. In addition to D. melanogaster and C. elegans*,* the modENCODE data also includes information collected from seven other drosophila and four other caenorhabditis species. To bring order to this diverse data set, the DCC built a multi-step pipeline to automate the submission and validation process. This pipeline uses controlled vocabularies to describe both project metadata and experimental protocols. The pipeline then collects and links submitted data together based on these common controlled vocabularies and executes a semi-automatic QC process to ensure the consistency and completeness of the submitted data.

**Figure 1:**
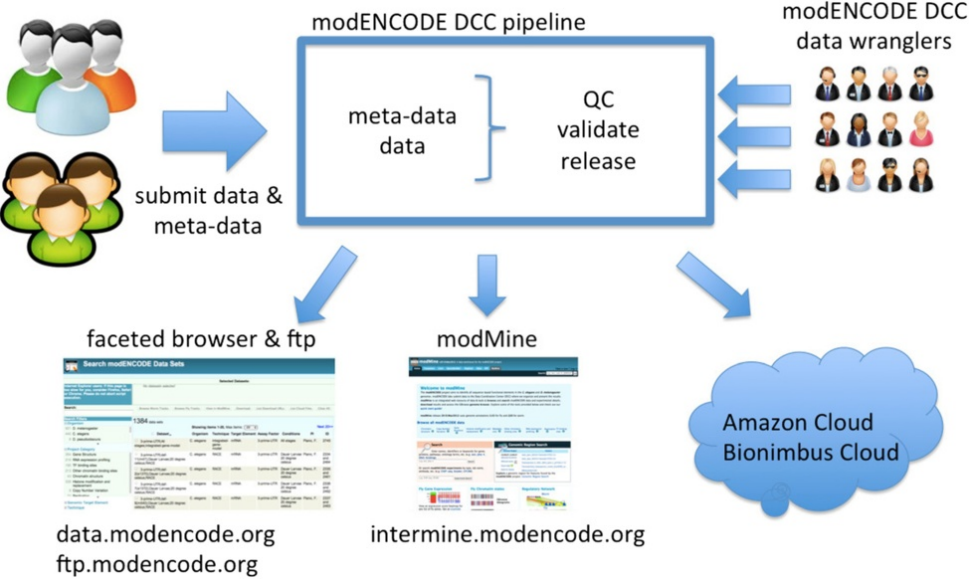
modENCODE DCC data flow from submission to release.

Once both metadata and data pass QC, they are released for use by the public. Figure 1 shows the data flow of the modENCODE DCC pipeline from data submission to data release. Table 1 lists the number and type of data sets released by modENCODE.

**Table 1:** modENCODE data sets, by category

**Category**	**Count**
Chromatin structure	50
Copy Number Variation	16
Gene Structure	425
Histone modification and replacement	475
Metadata only	4
Other chromatin binding sites	641
RNA expression profiling	930
Replication	39
TF binding sites	363
**Total**	**2,943**

## Implementation

The modENCODE DCC provides public access to the modENCODE data set in a variety of formats, and is also charged with releasing convenient tools for manipulating the data. With respect to data availability, all data released by modENCODE is available for bulk download from data.modencode.org, and for interactive browsing and mining from intermine.modencode.org. In addition, we have made the complete data set available on Amazon cloud (http://aws.amazon.com/ec2) as snapshots (http://ftp://ftp.modencode.org/DATA_SNAPSHOTS.txt) and on the Bionimbus cloud (http://www.bionimbus.org) as a shared directory (/glusterfs/data/modencode/modencode-dcc). Both cloud environments allow users to compute over the complete 10 TB of data without first having to download it to a local compute resource.

For a single user, although Galaxy can easily be installed and used locally, (http://wiki.galaxyproject.org/Admin/Get%20Galaxy), there are two major challenges: (i) to setup each of the tools and supporting data types; (ii) to configure Galaxy to work with a local compute cluster provided one is available. These procedures can take an estimated one to two weeks to complete. Additionally, transferring multiple terabytes of data from Amazon Cloud to the local Galaxy will take a significant amount of time and may require additional storage. Without a cluster, processing large quantities of data using just a local Galaxy server is not practical due to the amount of time necessary for analysis to complete. Using Galaxy on Amazon Cloud is an effective alternative as disk space and compute resources can be allocated and terminated dynamically as needed.

The Amazon cloud is pay-as-you-go, while Bionimbus is an academic cloud environment that is available to researchers via an application process (https://www.opensciencedatacloud.org/apply). A getting-started guide for accessing the data set on Amazon can be found at http://data.modencode.org/modencode-cloud.html.

Galaxy offers bioinformatics analysis *reproducibility*. Any analysis run by a user should be reproducible by another user. Galaxy supports this feature via ’workflows’, which users can create and share with each other. Each workflow is a sequence of one or more analysis steps with a pre-defined set of parameters and a fix number of inputs. The initial release of Galaxy workflows [3] for modENCODE has focused on peak calling in ChIP-seq data, taking advantage of the fact that the ENCODE and modENCODE projects have jointly adopted a uniform peak calling pipeline that provides uniform analysis of human, mouse, worm and fly. The ChIP-seq workflows use uniform input and output formats, making them easy to use and simplifying the process of performing comparisons among data sets, across species, and between public and private data. The ChIP-seq tools are available for public use in the Galaxy toolshed (http://toolshed.g2.bx.psu.edu/view/modencode-dcc) and are also preinstalled on the Amazon modENCODE Galaxy image described in a later section.

As of February 2013, the following DCC-provided ChIP-seq analysis tools were publicly available in the Galaxy toolshed:

•macs2 - model-based analysis of ChIP-Seq (version 2.0.10.2), written by Tao Liu (https://github.com/taoliu/MACS)

•PeakRanger - multi-purpose, ultrafast ChIP-Seq peak caller, written by Xin Feng (http://ranger.sourceforge.net/)

•IDR - Reproducibility and automatic thresholding of ChIP-seq data, written by Anshul Kundaje (https://sites.google.com/site/anshulkundaje/projects/idr)

•SPP - cross-correlation analysis package (http://code.google.com/p/phantompeakqualtools/)

•bamedit - merging, splitting, filtering, and QC of BAM files, written by Ziru Zhou

The choice of peak calling tools reflects discussions among the ENCODE and modENCODE data analysis working groups. The uniform peak calling pipeline adopted by these groups uses macs2 for calling broad peaks such as histone marks, SPP for calling punctate peaks such as transcription factor binding sites, and IDR to rank peaks based on reproducibility among replicates. PeakRanger was used during early iterations of the uniform peak calling pipeline, but was eventually dropped. Its main feature is superior performance and software stability, making it useful for a “quick look” at large genomes that would otherwise take a long time to process using the standard peak callers.

For each of the tools in the Galaxy toolshed, we provide full instructions on how to install and use these tools in Galaxy [4]. Except for a few minor changes in the SPP source codes^a^, the same versions of all the tools are installed on the cloud machine images and on Galaxy. This provides consistency across platforms regardless of which platform users choose to use for their analysis. Update of any of the tools will be done in all platforms to maintain consistency. All previous versions of machine images, tools, and wrappers on Galaxy are kept for backward-compatibility.

We have built Galaxy workflows for calling peaks and performing consistency analysis across both 2- and 3- replicate ChIP-seq experiments. This greatly reduces the complexity of executing peak calling. For example, the standard modENCODE uniform peak calling pipeline, which begins with raw FASTQ files and ends with peak call BED files, involves 39 analysis steps among four software tools. The corresponding Galaxy workflow can be executed in just a few clicks to select the input files and the species under consideration. These workflows, along with instructions on how to use them, are available on modENCODE DCC GitHub [4]. We encourage users to use these workflows and fine-tune parameters of tools in the workflow as they see fit.

### Launching modENCODE galaxy on amazon cloud

The modENCODE Galaxy instance may be launched as a single virtual machine, or as a cluster of machines that work together to speed up computations; in the latter case, the Galaxy user and admin interfaces run on the Galaxy instance, and the actual computation is done on a series of “worker nodes.” To run Galaxy on the Amazon public cloud, users must have an Amazon Web Services (AWS) account, and the credentials needed to launch instances in the Elastic Compute Cloud (EC2). To simplify the process of creating and configuring one or more Galaxy instances, we provide the Perl script *modENCODE_galaxy_create.pl* and its EC2 API command line tools dependencies, which are all available from our modENCODE Galaxy GitHub https://github.com/modENCODE-DCC/Galaxy, in the “bin” directory*.* To run this script, users will need Perl installed on their local machine (installed by default on most Linux distributions, and available from http://www.perl.org/get.html for Windows and MacOSX platforms). Prior to running the script the first time, users will need to modify the accompanying configuration file to hold their EC2 credentials, and preferences regarding the type, number and location of the Galaxy instances they wish to run. In addition to its basic function of launching Galaxy clusters in the Amazon cloud, the *modENCODE_galaxy_create.pl* script also provides status and location information for the launched instances, information on logging into the instance, and convenient tagging of all resources used by the Galaxy cluster.

**Figure 2:**
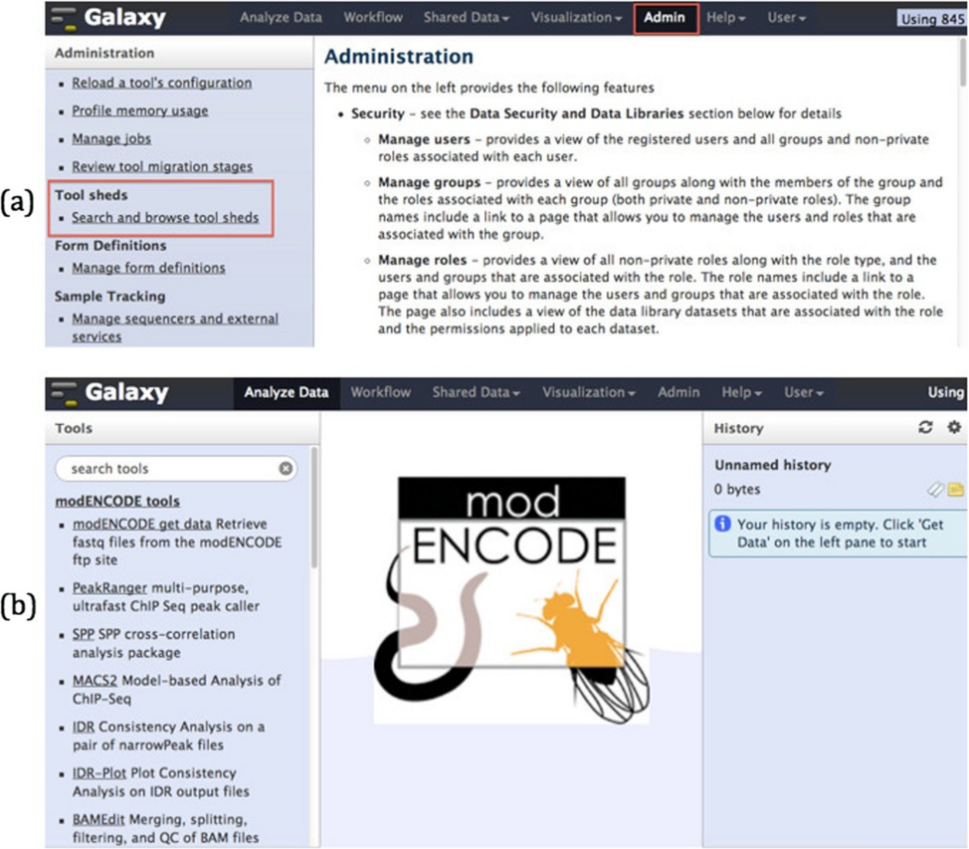
**(a) modENCODE tools can be installed via Galaxy administrator interface by clicking on ‘Admin’ and ‘Search and browse tool sheds’ (indicated by red boxes). (b) modENCODE Galaxy after installations of modENCODE tools and their dependencies.**

Once Galaxy is up and running, users must install the modENCODE tools onto their Galaxy cluster in either of two ways: (i) from toolshed via the Galaxy graphical administration interface; or (ii) by running the auto install script from the command line to install all of the modENCODE tools at once. Using the Galaxy administration interface, tools can be installed one by one as needed (Figure 2 panel (a)). This is suitable if users need only a handful of tools from the modENCODE tools collection. However, we recommend installing all the modENCODE tools so that multi-step workflows, such as the uniform peak calling pipeline, will run successfully.

The *auto_install.sh* script, which is also found in modENCODE Galaxy GitHub in the “bin” directory, takes about 10 minutes to complete as the downloading and installing are done in parallel. The *auto_install.sh* script also keeps track of worker nodes with tools downloaded and installed, so when one or more new worker nodes are added to the cluster, *auto_install.sh* will only download and install tools on these new additional computing nodes. Complete detailed and step-by-step instructions on how to launch Galaxy on Amazon Cloud and how to install tools on Galaxy are available on our GitHub [4]. Figure 2 panel (b) shows modENCODE Galaxy after installations of tools and their dependencies. Tools put together by modENCODE DCC are listed under ‘modENCODE tools’ menu in the tools panel. Tutorials on how to run these tools are also available in [4].

**Figure 3:**
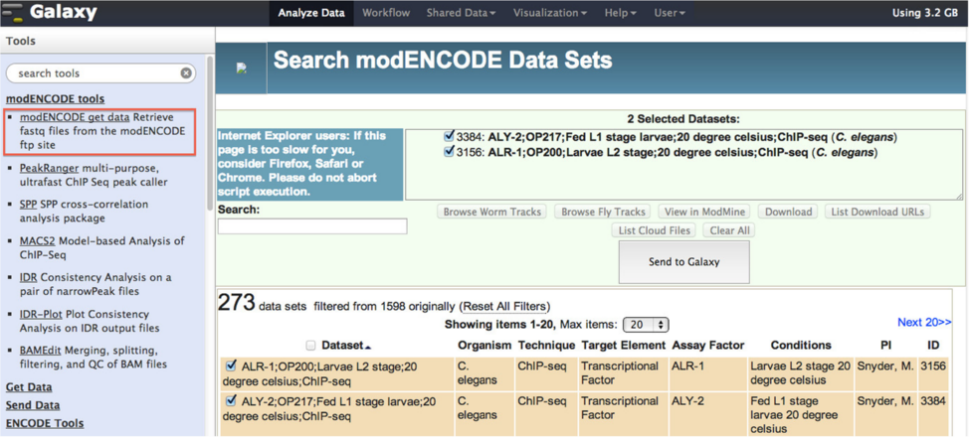
Data can be imported directly into Galaxy from our faceted browser.

Figure 3 shows one of the main features of modENCODE Galaxy, which allows users to use our faceted browser, search for the data sets they want to use and then import the results directly into their Galaxy for further analysis.

## Results

The uniform peak calling workflows take a FASTA reference genome and either 2 or 3 pairs of FASTQ files representing the ChIP and control (input) experiments. The workflows then perform the following steps:

•Convert each of the input FASTQ files to Sanger format if it is not already in Sanger format (http://en.wikipedia.org/wiki/FASTQ_format)

•Use bwa [5] to align each of the Sanger FASTQ files against the FASTA reference genome. Output files are in SAM format (http://samtools.sourceforge.net/SAM1.pdf)

•Convert SAM format output files to BAM format files

•Use macs2 to call peaks on each pair of ChIP and control BAM files

•Use IDR to compare the consistency between the replicates, for example, replicate 1 vs. replicate 2

•Use IDR-Plot to plot the consistency analysis from the previous step

This section shows how to run the 2-replicate uniform processing/peak calling workflow starting from the raw FASTQ files. For convenience, below are URLs that can be used to obtain the raw FASTQ files, reference FASTA file, and a 2-replicate workflow that were used in this exercise:

# ChIP and Input FASTQ files for replicates 1 and 2

http://data.modencode.org/modENCODE_Test_Data/fastq/3381/3381_Snyder_DAF-12_GFP_L3_rep1_ACGT.fastq.gzhttp://data.modencode.org/modENCO'DE_Test_Data/fastq/3381/3381_Snyder_DAF-12_Input_L3_rep1_ACGT.fastq.gzhttp://data.modencode.org/modENCODE_Test_Data/fastq/3381/3381_Snyder_DAF-12_GFP_L3_rep2_TGCT.fastq.gzhttp://data.modencode.org/modENCODE_Test_Data/fastq/3381/3381_Snyder_DAF-12_Input_L3_rep2_TGCT.fastq.gz

# worm reference genome WS220

http://data.modencode.org/modENCODE_Galaxy/Test_Data/fasta/WS220.fasta

# 2-replicate uniform workflow

http://data.modencode.org/modENCODE_Galaxy/workflows/Galaxy-Workflow-2-rep-peak-call-bwa-macs2-idr.ga

**Figure 4:**
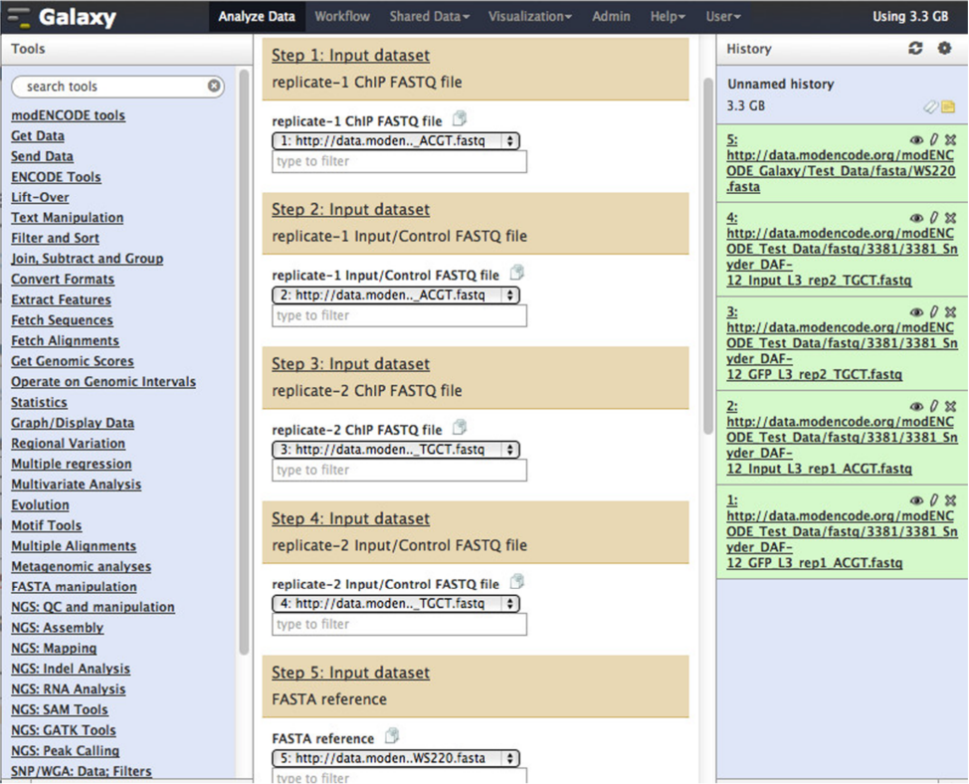
Running the 2-replicate uniform processing/peak calling workflow.

1. From the Galaxy instance’s web-based user interface, import the above FASTQ and FASTA files by clicking on ‘Get Data’ and ‘Upload File’. Cut and paste the above URLs into the URL/Text box and click on ‘Execute’. Once the uploaded is done, there should be 5 entries in the History panel as shown in Figure 4.

2. Import the 2-replicate uniform processing/peak calling workflow by clicking on ‘Workflow’ and ‘Upload or import workflow’

3. Once the workflow is uploaded, click on the workflow and select ‘Run’. Select the input data for the workflow as follows:

a. Step 1 input dataset: data entry 1 ( replicate 1 ChIP )

a. Step 2 input dataset: data entry 2 ( replicate 1 input/control )

a. Step 3 input dataset: data entry 3 ( replicate 2 ChIP )

a. Step 4 input dataset: data entry 4 ( replicate 2 input/control )

a. Step 5 input dataset: data entry 5 ( WS220 FASTA )

4. Execute the workflow by clicking on ‘Run workflow’.

Depending on the load, the workflow should take 4 to 6 hours to complete. Some of the important files output by the workflow are:

•Aligned files produced by bwa in bam format

•narrowPeak files produced by macs2 (http://genome.ucsc.edu/FAQ/FAQformat.html#format12)

•Overlapped peaks between the two replicates and above threshold generated by IDR

•The last step in the workflow is to produce a result PDF file, which shows the consistency between the two replicates.

**Figure 5:**
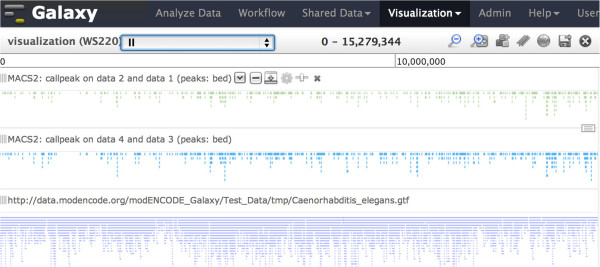
Galaxy visualization of peak call output for chromosome II from the workflow for the two sample replicates.

Figure 5 shows the visualization of peak output files from the workflow. The top and middle tracks show chromosome II peaks for Chip vs. Input for replicate 1 and 2. The bottom track is the gene annotation in GTF format for C. elegans.

To run the same workflow on your own data, simply use the Galaxy upload features to load your own set of ChIP/control FASTQ files. This will provide you with peaks that have been called and QC checked with methods identical to those used adopted by the modENCODE and ENCODE projects.

### Amazon cloud & bionimbus cloud

Uploading/downloading large data results to/from Galaxy via its graphical user interface can be slow (roughly 20× slower than a direct FTP transfer); furthermore, when a Galaxy cluster is terminated, all its data and results are deleted. Experienced users may prefer doing all analysis via the Unix command line rather than using Galaxy. To accommodate these users, we have created machine images on both the Amazon Public Cloud and the Bionimbus Community Cloud containing all the tools needed for the ENCODE/modENCODE uniform processing/peak calling pipeline. To use these images, researchers simply launch them, log into them via the secure shell, and run the pipeline using the provided scripts. Because both the Amazon and Bionimbus cloud images come with a complete copy of the modENCODE data set, there is no need to copy the data before starting to work with it. Step-by-step documentations on how to use either Amazon Cloud or Bionimbus Cloud are available at GitHub [4].

### Other online resources provided by modENCODE DCC

Other modENCODE computing resources available publicly are modMine, the faceted data browser and download server, and GBrowse. All can be reached from the modENCODE project portal at http://www.modencode.org.

#### modMine (http://intermine.modencode.org)

The modMine database and web interface [6] are based on the InterMine data warehousing system [7] to provide researchers with a powerful infrastructure to query the modENCODE metadata and data. Data produced by the 11 sub-projects are integrated with information from other public data sources in order to increase their utility. For instance, by including mappings to orthologous genes in other organisms [8], the opportunity to carry out comparative studies is provided. Other external data sources integrated in modMine included: genome annotations from WormBase [9] and FlyBase [10], Gene Ontology (GO) annotations [11], physical and genetic interactions [12,13], protein information [14], and protein domains [15].

Apart from its ability to integrate data from multiple sources, modMine has other useful features: (i) the ability to work with lists (for example, list of genes or list of submission identifications) and to provide basic analysis for them (GO terms and publications enrichments, chromosomal distributions, *etc.*); (ii) to access a library of commonly used search tasks available as ‘search templates’; (iii) to be able to extract data from a defined list of chromosomal locations (’Region Search’); and (iv), keyword searching with logical operations and faceted results. ModMine also provides extensive web-services and code-generation, which users can utilize to extract data programmatically from modMine directly to their local computers. Documentation and tutorials on how to use modMine are available from http://www.modencode.org.

#### Faceted data browser and download server (http://data.modencode.org &http://ftp://ftp.modencode.org)

The faceted browser (data.modencode.org) is a lightweight web-based application that allows users to search and explore modENCODE data by applying a combination of filters using a shopping catalogue metaphor. The filter system gives the user a flexible and intuitive mechanism for visualizing the diversity of data sets available, and narrowing them down to the subset that is relevant to his or her work. Currently, the following filters are available on the faceted browser: *Organism*, *Project Category*, *Genomic Target Element*, *Technique*, *Principle Investigator*, *Assay Factor*, *Developmental Stage*, *Strain*, *Cell Line*, *Tissue*, *Compound*, and *Temperature*. Each filter displays the number of data sets that would be selected if that filter is turned on, and the effects are interactive. For example, after we select “RNA-seq” from *Technique,* and “D. melanogaster” from *Organism,* then the *Development Stage* filter shows that there are 11 Adult Female experiments, 7 Adult Male experiments, 91 Late Embryo experiments, and so forth. When a search is performed or when one or more filters are selected, the faceted browser displays its results (both metadata and data) in a uniform format thus making it straightforward to compare between data sets and/or across species. Furthermore, once the list of data sets is narrowed down to the user’s satisfaction, he or she can press one of the navigational buttons to send the results to external applications such as Galaxy, GBrowse, modMine, or to create an archive to download the data files to their local computers.

Traditional FTP access to the modENCODE data set is available from http://ftp://data.modencode.org. Directories on the ftp server are organized into species and further divided into different data categories and data types. At the lowest level of directories are data files. All data files follow a common naming pattern, which is described in detailed at http://ftp://data.modencode.org/README.

It is also possible to launch a personal instance of the faceted browser and ftp server within the Amazon Cloud, letting users create customized instances of these services. Information on how to do this is available at http://data.modencode.org/modencode-cloud.html.

#### Generic Genome Browser (GBrowse) (http://modencode.oicr.on.ca/fgb2/gbrowse/worm &http://modencode.oicr.on.ca/fgb2/gbrowse/fly)

All level 2 modENCODE data sets can be browsed and analyzed in a genome browser using the GBrowse 2 (http://gmod.org/wiki/GBrowse) software. The browser offers track-level configuration, stable user accounts, and a data track uploading facility. In addition to using the public modENCODE GBrowse instance, advanced users can run a copy of the genome browser server within the Amazon cloud, thereby allowing them to host a complete copy of the modENCODE data set and to add their own private data sets to the corpus. Instructions for doing this can be found at http://data.modencode.org/modencode-cloud.html.

## Conclusions

We provided ready-to-use tools and workflows on Galaxy for the uniform processing/peak calling pipeline, which has been adopted by both the modENCODE and ENCODE communities. Using the resources we put together not only save users time from installing tools and reference data but also provides consistent and reproducible analysis results. Our tools and workflows are designed to work with human, mouse, worm, and fly genomes and thus cross species comparisons are possible. For advanced users, we also provide machine images on both Amazon and Bionimbus Clouds so the same consistent and reproducible analysis can be done from the command line.

## Future work

Although the worm and fly genomes are relatively small, the ENCODE/modENCODE uniform processing/peak calling pipeline may take several hours to complete on a single machine. The human and mouse genomes, which are over 20× larger, take proportionately longer to run. To speed up the analysis, we are parallelizing the tools in the Galaxy-based ENCODE/modENCODE uniform processing/peak calling pipeline so that the work can be split among multiple worker nodes. Similarly, we are adding support for Sun Grid Engine to the Amazon and Bionimbus modENCODE machine images, thereby simplifying the task of running the pipeline on a virtual compute cluster.

## Availability and requirements

**Project name:** modENCODE Galaxy

**Project homepage:**https://github.com/modENCODE-DCC/Galaxy

**Operating system(s):** Unix

**Programming language:** Perl

**Other requirements:** EC2 API tools, Galaxy Amazon Machine Image (AMI)

**License:** all modENCODE Galaxy scripts are available free of charge to academic, government, and non-profit institutions.

## Endnote

^a^Changes in SPP source code are mainly: (i) to make SPP work on Galaxy; (ii) to generalize parameters of SPP so that it works with all four species (human, mouse, worm, and fly).

## Abbreviations

DCC: Data Coordinating Center; IDR: Irreproducible Discovery Rate; GO: Gene Ontology; modENCODE: Model Organism ENCyclopedia of DNA Elements; NIH: National Institutes of Health; QC: Quality Control.

## Competing interests

The authors declare that they have no competing interests.

## Authors’ contributions

QMT and LDS are responsible for the conception, design, and writing of the manuscript. FJ, ZZ, and KMC are responsible for the designs and implementations of scripts to launch Galaxy on Amazon Cloud and configurations of tools on Galaxy toolshed. MDP is responsible for the curation of modENCODE data. ETK is responsible for the main modENCODE pipeline. SC is responsible for the development and maintaining of modMine. PR is responsible for the development and maintaining of GBrowse. All authors read and approved the final manuscript.
